# Mass Trapping *Drosophila suzukii*, What Would It Take? A Two-Year Field Study on Trap Interference

**DOI:** 10.3390/insects13030240

**Published:** 2022-02-28

**Authors:** Rik Clymans, Vincent Van Kerckvoorde, Tom Thys, Patrick De Clercq, Dany Bylemans, Tim Beliën

**Affiliations:** 1 Zoology Department, Research Centre for Fruit Cultivation (pcfruit npo), Fruittuinweg 1, B-3800 Sint-Truiden, Belgium; rik.clymans@pcfruit.be (R.C.); vincent.vankerckvoorde@pcfruit.be (V.V.K.); tom.thys@pcfruit.be (T.T.); dany.bylemans@pcfruit.be (D.B.); 2 Department of Plants and Crops, Faculty of Bioscience Engineering, Ghent University, Coupure Links 653, B-9000 Ghent, Belgium; patrick.declercq@ugent.be; 3 Department of Biosystems, KU Leuven, Decroylaan 42, B-3001 Heverlee, Belgium

**Keywords:** *Drosophila suzukii*, trap competition, semiochemicals, trap density, attraction radius, *Prunus cerasus*, attract and kill, controlled release dispensers, apple cider vinegar, fruit flies

## Abstract

**Simple Summary:**

*Drosophila suzukii* is an invasive fruit fly that have became a key pest of soft-skinned fruits during the past decade. Today, the control of this pest relies strongly on broad-spectrum insecticides. Deploying attractive traps to control the pest population (mass trapping) could be part of the management strategy of *D. suzukii.* The present study analyses whether mass trapping with different attractants could be viable for *D. suzukii* control and how far traps should be maximally spaced in a grid. Traps in a grid compete for the same insects when they are spaced close enough and their radii of attraction overlap. Since the traps on the corners of a grid have fewer competing traps around than fully surrounded centre traps, the ratio of the catches in the corner traps and the centre traps indicates whether the traps are spaced close enough. By quantifying that trap interference in 4 × 4 trapping grids, it was found in this two-year field study that workable trap densities can be expected to control *D. suzukii.* From June onwards, synthetic lures in dry traps show equal or better results than the same traps with a reference liquid bait (apple cider vinegar).

**Abstract:**

The invasion of *Drosophila suzukii* (Matsumura) (Diptera: Drosophilidae) worldwide has disrupted existing or developing integrated pest management (IPM) programs in soft-skinned fruits. Currently, with a reliance on only broad-spectrum insecticides, there is a critical call for alternative control measures. Behavioural control is one of the pillars of IPM, and, in the present study, it is investigated whether mass trapping could be viable for *D. suzukii* management. By quantifying trap interference in 4 × 4 replicate trapping grids, an estimate of the attraction radius for a certain attractant and context can be obtained. Traps designed for dry trapping (no drowning solution, but a killing agent inside) and synthetic controlled released experimental lures were tested in a two-year field study. Apple cider vinegar (ACV) was included as a reference bait and trials were performed with 5, 10 and 15 m inter-trap spacings at different seasonal timings. Clear trap interference and, hence, overlapping attraction radii were observed both in spring and summer for both the synthetic lures and ACV. In early spring, ACV shows the most potential for mass trapping, however from June onwards, the experimental dry lures show equal or better results than ACV. Based on our findings, workable trap densities are deemed possible, encouraging further development of mass trapping strategies for the control of *D. suzukii*.

## 1. Introduction

The spotted wing Drosophila, *Drosophila suzukii* Matsumura (Diptera: Drosophilidae), native to Asia [1,2,3], became a worldwide invasive key pest of soft-skinned fruits over the last decades [1,4,5,6,7,8,9,10,11]. As *D. suzukii* is a serious economic pest [12,13,14,15], its invasion forced fruit growers to revert to calendar-based sprays with (broad-spectrum) insecticides [1,13,16,17]. In the last decade, there have been numerous research efforts to devise alternative control measures and hence, to develop integrated pest management (IPM) programmes [18].

Traps are an important tool for IPM, as they can be used for monitoring and/or control by mass trapping [19]. Much of the research focused on fermented food baits, based on vinegars, wine, baker’s yeast, sugar, flour, proteins, molasses, etc. [19,20,21,22,23,24,25,26,27,28,29,30,31,32], with apple cider vinegar (ACV) still being a reference bait. Additionally, the attraction to volatiles produced by symbiont micro-organisms of *D. suzukii* [33,34,35,36,37,38,39,40,41] and host plant/fruit volatiles has been investigated for the development of *D. suzukii* lures [42,43,44,45,46,47,48,49,50]. The identified attractive chemicals have been tested by adding them to known baits [24,34] or fruit juices [51] or have been applied in controlled release dispensers [24,52,53,54,55,56,57,58,59]. The design of the traps is also well studied, considering visual cues, such as colour [60,61,62,63,64,65] and shape [20,28,60,65,66,67] as well as fly retention mechanisms [21,67,68,69]. *D. suzukii* populations are known to shift olfactory preferences throughout the year [70], so it is important to test attractants at different (relevant) seasonal timings.

Both for monitoring and mass trapping, liquid baits or drowning solutions are inconvenient as they need timely bait replacement [67,69,71] and hinder fly identification by the need for sieving and the presence of microbial growth or staining, etc. Sticky surfaces (insect glue), on the other hand, also can complicate species identification (monitoring) [69], can show ineffective retention [68,72] and can quickly become saturated. Traps with a controlled release lure and a killing agent on the inside have shown to significantly increase fly retention for *D. suzukii* [68] and are considered ideal for mass trapping dipteran pests as they require minimal labour (merely deploying and retrieving if the lure longevity is adequate) [73].

Although mass trapping *D. suzukii* was already investigated in Japanese cherry orchards in the 1930s [25], it has since been relatively understudied. Moreover, the few available studies use rather few replicates of small plots [21,74,75], which can cause interference with other treatments and maximize boundary effects. For mass trapping insects, it is key that it can be achieved at an economically feasible trap density. Investigating mass trapping control efficacy and “dose (density) finding” is not straightforward, as for most semiochemical-based control measures (e.g., mating disruption, male annihilation technique and attract-and-kill), they either require large replicate plots [76,77,78,79,80] and/or specialised experimental methods. Mark–release–recapture (MRR) studies are part of the specialised methods suitable for *D. suzukii*, with effective marking methods for this pest being available [81,82]. MRR studies and studies based on the relation between catches of attractive and passive traps can provide parameters for modelling capture probabilities in a specific trapping grid [83,84,85,86,87,88,89]. Another specialised method of estimating the potential of a certain trap and attractant for mass trapping a pest is to assess the interference between traps within a spatial pattern [90,91,92,93,94,95].

Building on that earlier work, Suckling et al. (2015) developed a relatively simple theory and method using square 4 × 4 minigrids for the quantification of trap competition and hence, the level of overlap of the attraction radii of the traps. Insects can be trapped when they occur within the attraction radius (r) of a trap, with attraction radius being the combined effect of the dispersal of the insect and the lure’s plume formation. When the targeted insects occur uniformly in the field, traps deployed in a square grid can compete for the same insects if their attraction radii overlap. Thus, the relation of the attraction radius (r) and the inter-trap spacing (d) determines whether these traps are competing. If the traps are deployed at an inter-trap spacing double or more than the attraction radius (d/r ≥ 2), they will not compete and hence not influence each other’s trap catches (i.e., no interference). The more the traps in the grid overlap in attraction radius, the stronger the competition among the traps. Then, it also becomes apparent that there should be within grid positional interference. In a 4 × 4 minigrid, the four traps located on the corners of the grid are least affected by trap interference as they have fewer competing traps surrounding than the four fully surrounded traps in the centre. The eight traps on the edges experience less catch suppression than the centre traps, but substantially more than the corner traps. Assessing the insect catches by the traps of a 4 × 4 minigrid enables to quantify the corner: centre trap catch ratio, which can provide an estimate of the attraction radius. A numerical simulation model of the relation between the ratio of corner: centre trap catches and the trap spacing relative to the attraction radius (d/r) was developed by Suckling et al. (2015). It can be assumed that, for mass trapping, the area must be completely covered by the attraction radii of the trapping grid. In a square grid this means that d/r should be smaller than $\sqrt{2}$ (overlap of diagonally neighbouring traps) [94].

The attraction radius of a trap depends on the effect that its attractant has on the target insect, this is, being influenced by the insect’s seasonal or life-history specific sensitivity and preferences. The attraction also may vary as a function of the climatic effects on the volatilisation, plume formation of attractants and, in the case of a fermenting baits, on the microbial activity. Therefore, in the present two-year study, 4 × 4 minigrids are used to quantify trap interference at different inter-trap spacings (5, 10 and 15 m) and seasonal timings (spring (May–June) and summer (August–September)). All are tested in the same (non-sticky, killing agent containing) trap type; two experimental dry synthetic lures are compared with ACV, a liquid bait.

This is the first focused study on the potential of mass trapping *D. suzukii.* By gaining insights in the attraction radii of different attractants at different seasonal timings, this study might contribute to the development of a cost-effective mass trapping system for *D. suzukii.*

## 2. Materials and Methods

### 2.1. Traps and Attractants

Over all trials, one single trap design was used, regardless of the lures or bait. This trap was Decis^®^ Trap Suzukii (Bayer Crop Science, Monheim, Germany): a red spherical trap with tunnel entries and a transparent lid, coated on the inside with deltamethrin. Van Kerckvoorde et al. (2020) specified the technical details and advantages of this particular trap design [68]. As a reference attractant, pure apple cider vinegar (ACV, 5% acidity, Burg, Vinaigrerie Fuchs, La Tremblade, France) was used. Each ACV baited trap contained 200 mL of the liquid, which was replaced weekly. In the trials of 2018, next to ACV, an experimental lure (EL1) was used; this lure, based on synthetic kairmones in controlled release dispensers in combination with the killing-agent-containing trap design, constituted a dry, non-sticky *D. suzukii* trap. In the trials of 2020, in addition to ACV and EL1, a second and similar experimental lure (EL2) was included.

### 2.2. Trap Interference Trials

#### 2.2.1. Spring 2018―5 m Inter-Trap Spacing 

In a 6 ha commercial orchard of sour cherry (*Prunus cerasus* cv. Kelleriis, row spacing of 6 m and within-row plant distance of 5 m, fully grown trees with canopy height 5 to 6 m and width of 4.5 m, 50°46.693′ N, 5°15.136′ E, surrounded by arable crops and a sweet cherry orchard, Sint-Truiden, Belgium), a trap interference trial was performed in the spring of 2018. In this trial, trap competition was quantified for traps hanging at what can be considered minimal inter-trap spacing: one trap per tree. The 16 traps in the 4 × 4 minigrids were thus suspended at about 5.5 m (trees planted 5 × 6 m) from each other. Both attractants, ACV and EL1, were tested under these conditions in four replicate minigrids. These eight minigrids were maximally spaced out in the orchard resulting in a minimal distance of 50 m between the grids and a minimal distance of 40 m between any grid and the orchard’s edges. The attractants were randomly assigned to the minigrids. The traps were deployed at the end of flowering on 27 April, suspended at 1.5 m height, near the centre of the tree. All traps were emptied weekly in individual containers and the content was (for ACV after sieving) stored in >80% ethanol. The weekly sampling was continued until 28 June, which was six days before mechanical harvest. Therefore, the trial had a total trapping period of nine weeks. The insecticide treatments during the trial were with acetamiprid on 10 June, spinosad on 17 June and lambda-cyhalothrin on 22 June and were homogeneously applied to the whole orchard. During the first 5 weeks of this trial (~May), there was a mean temperature of 15.76 °C (T_min_ = 1.4 °C, T_max_ = 31.1 °C), relative humidity (RH) of 69.49%, and windspeed of 3.36 m/s with a mean wind direction of 42.8°, i.e., northeast (NE). During the last 4 weeks of this trial (~June), there was a mean temperature of 17.63 °C (T_min_ = 4.8 °C, T_max_ = 29.9 °C), RH of 75.09%, and windspeed of 3.10 m/s with a mean wind direction of 314.5°, i.e., northwest (NW). Over the whole test period (i.e., May and June together) the mean wind direction was 334.7°, i.e., north–northwest (NNW).

#### 2.2.2. Summer 2018―5 m Inter-Trap Spacing

In an 8 ha commercial orchard of sour cherry (*Prunus cerasus* cv. Kelleriis, row spacing of 6 m and within-row plant distance of 4 m, fully grown trees with canopy height 5 to 6 m and width of 4.5 m, 50°43.548′ N, 5°13.703′ E, surrounded by arable crops, pome fruit orchards and a small forest fragment, Gingelom, Belgium), first the same trial from spring 2018 was repeated under summer conditions. The 16 traps in the 4 × 4 minigrids were hung at about 5 m (trees planted 4 × 6 m) from each other. The same attractants (ACV and EL1) were thus tested at a similar ~5 m inter-trap spacing, this time however during August, one month after harvest. Again, for each attractant, four replicate minigrids were installed, in this case with a minimal distance of 60 m between the grids and a minimal distance of 30 m between any grid and the orchard’s edges. Traps were deployed on 3 August and the following two weeks trap catches were collected and stored as mentioned above. Since the population size of *D. suzukii* in August is very high (due to reproduction on ripe and rotting cherries), two weeks were sufficient to obtain the desired sample sizes. During this trial, there was a mean temperature of 19.53 °C (T_min_ = 11.3 °C, T_max_ = 37.2 °C), RH of 74.93%, and windspeed of 4.17 m/s with a mean wind direction of 228.9°, i.e., southwest (SW).

#### 2.2.3. Summer 2018―10 m Inter-Trap Spacing

On 16 August the second summer trial was installed, simply by bringing the traps of the aforementioned grids to 10 m inter-trap spacing. As a consequence, the minimal distance between the grids became 40 m and the minimal distance between any grid and the orchard’s edges became 25 m. Now at 10 m, the same weekly sampling continued for three weeks with the last sampling on 6 September. There were no insecticide treatments after harvest in this orchard. During this trial, there was a mean temperature of 17.26 °C (T_min_ = 4.7 °C, T_max_ = 29.3 °C), RH of 79.74%, and windspeed of 2.96 m/s with a mean wind direction of 288.7°, i.e., west–northwest (WNW).

#### 2.2.4. Spring 2020―5 m Inter-Trap Spacing 

In the same 8 ha orchard (Gingelom, Belgium), the trial conducted in the spring of 2018 with a ~5 m inter-trap spacing was repeated in 2020, the only difference being that an improved lure (EL2) was added to the design. Thus, now 12 minigrids of 4 × 4 traps were installed: 4 replicates for each of the 3 attractants (ACV, EL1 and EL2). In this trial, there was a minimal distance of 50 m between the grids and a minimal distance of 30 m between any grid and the orchard’s edges. Traps were deployed in the same week as in 2018 (week 17, on 24 April) and their content was weekly collected for 10 weeks. As the harvest was later in 2020 (11 July), the traps were kept in field one week longer than in 2018. The insecticide treatments during the trial were with pirimicarb and lambda-cyhalothrin on 26 April, acetamiprid on 12 June and lambda-cyhalothrin on 3 July and were homogeneously applied to the whole orchard. During the first 5 weeks of this trial (~May), there was a mean temperature of 13.13 °C (T_min_ = −2.0 °C, T_max_ = 30.8 °C), RH of 56.82%, and windspeed of 3.66 m/s with a mean wind direction of 339.7°, i.e., north–northwest (NNW). During the last 5 weeks of this trial (~June), there was a mean temperature of 17.28 °C (T_min_ = 6.2 °C, T_max_ = 31.9 °C), RH of 63.36%, and windspeed of 3.92 m/s with a mean wind direction of 230.9°, i.e., SW. Over the whole test period (i.e., May and June together) the mean wind direction was 250.6°, i.e., west–southwest (WSW).

#### 2.2.5. Summer 2020―5 m Inter-Trap Spacing

In the summer of 2020, the trials from 2018 were also repeated with the addition of the improved lure (EL2). First, the same minigrids (5 m inter-trap spacing) as in the spring 2020 trial were reinstalled in the same sour cherry orchard (Gingelom, Belgium) on 4 August (3 August 2018). As in 2018, two-week trap catches were sampled. During this trial, there was a mean temperature of 23.6 °C (T_min_ = 12.2 °C, T_max_ = 38.4 °C), RH of 59.7%, and windspeed of 2.9 m/s with a mean wind direction of 163.4°, i.e., south–southeast (SSE).

#### 2.2.6. Summer 2020―10 m Inter-Trap Spacing

On 18 August, the second summer trial was installed, as in 2018 at 10 m inter-trap spacing, but with the additional EL2 lure. The minimal distance between the grids was 40 m and the minimal distance between any grid and the orchard’s edges was 25 m. Now at 10 m, the same weekly sampling continued for three weeks with the last sampling on 8 September. During this trial, there was a mean temperature of 16.8 °C (T_min_ = 6.9 °C, T_max_ = 33.5 °C), RH of 71.3%, and windspeed of 4.3 m/s with a mean wind direction of 231.5°, i.e., SW.

#### 2.2.7. Summer 2020―15 m Inter-Trap Spacing

An additional trial was performed in September 2020, with trap competition for lure EL2 being quantified for an inter-trap spacing of 15 m. Four replicates of the minigrid were installed on 8 September. This wider spacing impeded testing other attractants at the same time in this orchard. There was a minimal distance of 50 m between grids and between any grid and the orchard’s edges. For two weeks, trap catches were sampled. There were no insecticide treatments after harvest in this orchard. During this trial, there was a mean temperature of 17.8 °C (T_min_ = 4.9 °C, T_max_ = 36.6 °C), RH of 64.1%, and windspeed of 2.84 m/s with a mean wind direction of 85.2°, i.e., east (E).

### 2.3. Assessments

The collected content of each trap was labelled and stored in >80% ethanol. The content of ACV was sieved to remove the ACV drowning solution before adding ethanol. Per sample, male and female *D. suzukii* flies were counted under a binocular microscope. The samples of 2018 were all fully sorted as such. For the larger samples from the summer of 2020, a method of subsampling based on Elsensohn and Loeb (2018) [96] was adopted. First, large non-target insects were manually removed from the sample, and subsequently the sample was sieved to remove the storage ethanol. The underside of the sieve was dabbed with paper towelling until there was no free ethanol in the sample. The sample was weighed using a Sartorius Genius ME215P balance (Sartorius, Goettingen, Germany; precision: 0.1 mg) and if it was ≥2 g, a 10% (mass) subsample was randomly taken for assessment. Only 18% of the (1088) summer 2020 samples exceeded the 2 g threshold. This method was evaluated for accuracy: extrapolation (multiplying counts by 10) of these subsamples and afterwards counting the complete sample resulted in a mean difference of 5.76% (*n* = 5, SD = 3.75). Weather data were obtained from Mety (Bodata, Dordrecht, The Netherlands) weather stations within a 7 km and 1 km range of the experimental site for the spring 2018 trial and all other trials, respectively. Mean windspeed and wind direction during the trials were obtained for both locations from Weatherbit [97]. As wind direction is a circular variable with 0° and 360° representing the same direction (north), the mean wind direction over a period was calculated using *Mean wind direction (degrees) = arctan2(mean cos(wind direction), mean sin(wind direction))* ∗ (180/π)) [98].

### 2.4. Data Analysis

All statistical analyses were performed in R v3.6.3 [99]. In order to quantify trap interference between corner and centre traps, per trial all *D. suzukii* flies were pooled over the sampling dates per trap and per attractant and a generalized linear mixed model (GLMM) with Poisson distributed errors and a log-link function was used to model these fly counts in each trap. The position (corner or centre) was used as a fixed factor, while the exact trap location nested in the replicate grid was added as a random factor. The model’s appropriateness was assessed through diagnostic residual plots and evaluation of dispersion. Post hoc Tukey pairwise comparison was performed using the R *emmeans* package (version 1.4.5) at a minimum significance level α = 0.05. The comparison of the corner: centre ratios between attractants was performed by calculating the ratio per replicate grid from the pooled counts in the four corner traps over the pooled counts in the four centre traps over the whole trapping period. Hence, per trial, four replicate ratios per attractant were obtained. Differences in ratios between attractants were analysed using one-way ANOVA after the evaluation of the assumptions with post hoc Tukey pairwise comparison. Attractant trap capture was analysed with GLMM with Poisson distributed errors and a log-link function per sex per period (May, June and August) with *D. suzukii* counts pooled per trap over the whole period as a dependent variable, attractant as fixed and trap nested in replicate grid as random factor. The trials at 5 and 10 m inter-trap spacing in summer were pooled and labelled as period ‘August’ for this analysis.

## 3. Results

### 3.1. Trap Interference

#### 3.1.1. Spring—5 m Inter-Trap Spacing

**Figure 1:**
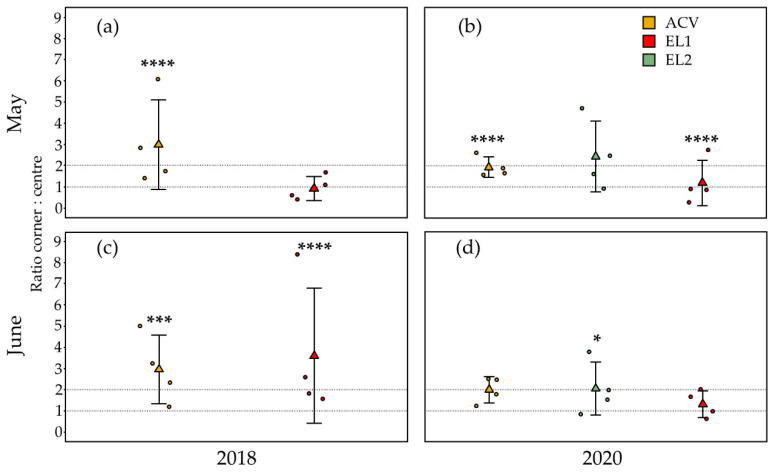
Corner to centre trap catch (*D. suzukii*) ratios for the two and three attractants tested in spring 2018 and 2020, respectively, at 5 m inter-trap spacing. Jittered points represent the ratio per replicate (*n* = 4) grid, triangles depict their mean and error bars are standard deviations. Asterisks above graphs refer to the level of significance regarding the difference in counts between corner and centre traps (*n* = 16) analysed for each attractant and period (GLMM, * ≤ 0.05, ** ≤ 0.01, *** ≤ 0.001, **** ≤ 0.0001). Between the attractants, no statistical differences were found in corner: centre ratios. (**a**) In May 2018, ACV had significantly more trap catches in the corner traps than in the centre traps. (**b**) In May 2020, a similar result was obtained for ACV, whereas EL1 had significant less trap catches in the corner traps than in the centre traps. (**c**) In June 2018, both ACV and EL1 had significantly more trap catches in the corner traps. (**d**) In June 2020, only EL2 had significantly higher trap catches in the corner traps than in the centre traps.

In the trial at 5 m inter-trap spacing in May 2018 (34 days, from 27 April to 31 May), there were significantly more *D. suzukii* flies caught in the corner traps than in the centre traps for ACV (GLMM: z-ratio = 4.85, *p* < 0.0001, *n* = 16) (Figure 1a). This was not the case for EL1 (GLMM: z-ratio = −0.22, *p* = 0.83, *n* = 16). The mean (*n* = 4) ratio of flies captured in the corner traps over flies captured in centre traps for ACV and EL1 was 2.99 and 0.93, respectively; these means were not statistically different. In the trial at 5 m inter-trap spacing in May 2020 (35 days, from 24 April to 29 May), there were significantly more *D. suzukii* flies caught in the corner traps than in the centre traps for ACV (GLMM: z-ratio = 5.23, *p* < 0.0001, *n* = 16) (Figure 1b). This was not the case for EL2 (GLMM: z-ratio = 1.90, *p* = 0.06, *n* = 16). Conversely, there were significant fewer (GLMM: z-ratio = −36.64, *p* < 0.0001, *n* = 16) flies caught in the corner traps with EL1 than in the centre traps. The mean (*n* = 4) ratio corner: centre for ACV, EL1 and EL2 in this period was 1.94, 2.44 and 1.20, respectively; these means were not statistically different.

In the trial at 5 m inter-trap spacing in June 2018 (28 days, from 31 May to 28 June), there were significantly more *D. suzukii* flies caught in the corner traps than in the centre traps for both ACV (GLMM: z-ratio = 3.28, *p* = 0.001, *n* = 16) and EL1 (GLMM: z-ratio = 4.63, *p* < 0.0001, *n* = 16) (Figure 1c). The mean (*n* = 4) ratio corner: centre for ACV and EL1 in this period was 2.96 and 3.60, respectively; these means were not statistically different. In the trial at 5 m inter-trap spacing in June 2020 (35 days, from 29 May to 3 July), there were significantly more *D. suzukii* flies caught in the corner traps than in the centre traps for EL2 (GLMM: z-ratio = 2.05, *p* = 0.04, *n* = 16) (Figure 1d). This was not the case for both ACV (GLMM: z-ratio = 1.92, *p* = 0.06, *n* = 16) and EL1 (GLMM: z-ratio = 0.19, *p* = 0.85, *n* = 16). The mean (*n* = 4) ratio corner: centre for ACV, EL1, and EL2 in this period was 2.01, 1.32, and 2.06, respectively; these means were not statistically different.

#### 3.1.2. Summer—5 m Inter-Trap Spacing

**Figure 2:**
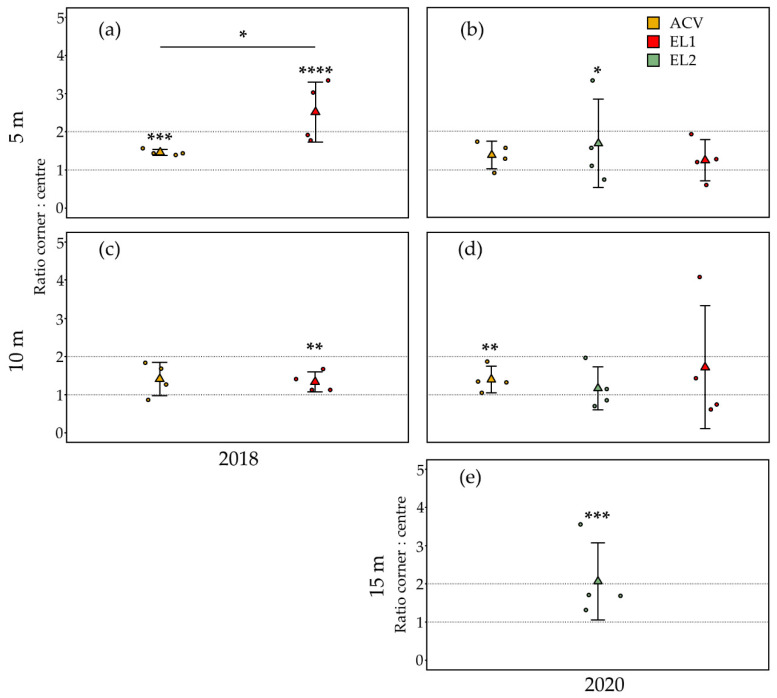
Corner to centre trap catch (*D. suzukii*) ratios for the two and three attractants tested in summer 2018 and 2020, respectively. Trials were conducted at an inter-trap spacings of 5 and 10 m, for EL2 15 m was tested as well. Jittered points represent the ratio per replicate (*n* = 4) grid, triangles depict their mean and error bars are standard deviations. Asterisks above graphs refer to the level of significance regarding the difference in counts between corner and centre traps (*n*= 16) analysed for each attractant and period (GLMM, * *p* ≤ 0.05, ** *p* ≤ 0.01, *** *p* ≤ 0.001, **** *p* ≤ 0.0001). Between the attractants, only a statistical difference was found in the corner: centre ratios in the trial at 5 m in 2018, shown by a line over the different attractants and an asterisk (ANOVA, F (1,6) = 7.18, *p* = 0.04). (**a**) At 5 m spacing in 2018, both ACV as EL1 showed significantly more trap catches in the corner traps than in the centre traps, but the mean corner: centre ratio of EL1 was significantly higher than that of ACV. (**b**) In 2020, at 5 m spacing, only EL2 showed significantly higher trap catches in the corner traps than in the centre traps. (**c**) When spacing the traps at 10 m in 2018, EL1 caught significantly more flies in the corner traps than in the centre traps, whereas ACV did not. (**d**) In 2020, at 10 m the opposite was observed: significantly higher trap catches in the corner traps than in the centre traps for ACV, but not for both experimental lures. (**e**) When spacing the EL2 traps 15 m in 2020, significantly more flies were caught in the corner traps than in the centre traps.

In the trial at 5 m inter-trap spacing in August 2018 (13 days, from 3 to 16 August), there were significantly more *D. suzukii* flies caught in the corner traps than in the centre traps for both ACV (GLMM: z-ratio = 3.40, *p* = 0.0007, *n* = 16) (Figure 2a) and EL1 (GLMM: z-ratio = 4.30, *p* < 0.0001, *n* = 16). The mean (*n* = 4) ratio of flies captured in the corner traps over flies captured in centre traps for ACV and EL1 in this trial was 1.46 and 2.52, respectively, the latter being significantly higher than the former (ANOVA, F (1,6) = 7.18, *p* = 0.04). In the trial at 5 m inter-trap spacing in August 2020 (14 days, from 4 to 18 August), there were significantly more *D. suzukii* flies caught in the corner traps than in the centre traps for EL2 (GLMM: z-ratio = 1.98, *p* = 0.048, *n* = 16) (Figure 2b), but this was not the case for both ACV (GLMM: z-ratio = 1.40, *p* = 0.16, *n* = 16) and EL1 (GLMM: z-ratio = 0.10, *p* = 0.92, *n* = 16). The mean (*n* = 4) ratio of flies captured in the corner traps over flies captured in centre traps for EL2 in this trial was 1.69. For ACV and EL1, it was 1.38 and 1.25, respectively; these three means were not statistically different.

#### 3.1.3. Summer—10 m Inter-Trap Spacing

In the trial at 10 m inter-trap spacing in August 2018 (21 days, from 16 August to 6 September), there were significantly more *D. suzukii* flies caught in the corner traps than in the centre traps for EL1 (GLMM: z-ratio = 2.73, *p* = 0.0063, *n* = 16) (Figure 2c). This was not the case for ACV (GLMM: z-ratio = 1.73, *p* = 0.09, *n* = 16). The mean (*n* = 4) ratio of flies captured in the corner traps over flies captured in centre traps for ACV and EL1 was 1.41 and 1.34, respectively; these means were not statistically different. In the trial at 10 m inter-trap spacing in August 2020 (21 days, from 18 August to 8 September), there were significantly more *D. suzukii* flies caught in the corner traps than in the centre traps for ACV (GLMM: z-ratio = 2.84, *p* = 0.005, *n* = 16) (Figure 2d), which was not the case for both EL1 (GLMM: z-ratio = 1.05, *p* = 0.30, *n* = 16) and EL2 (GLMM: z-ratio = 0.79, *p* = 0.43, *n* = 16). The mean (*n* = 4) ratio of flies captured in the corner traps over flies captured in centre traps for ACV, EL1 and EL2 was 1.40, 1.72 and 1.17, respectively; these three means were not statistically different.

#### 3.1.4. Summer—15 m Inter-Trap Spacing

In the trial of EL2 at 15 m inter-trap spacing in September 2020 (14 days, from 8 to 22 September), there were significantly more *D. suzukii* flies caught in the corner traps than in the centre traps (GLMM: z-ratio = 3.60, *p* = 0.0003, *n* = 16) (Figure 2e). The mean (*n* = 4) ratio of flies captured in the corner traps over flies captured in centre traps was 2.06.

### 3.2. Spatial Patterns in Trapping Grids

**Figure 3:**
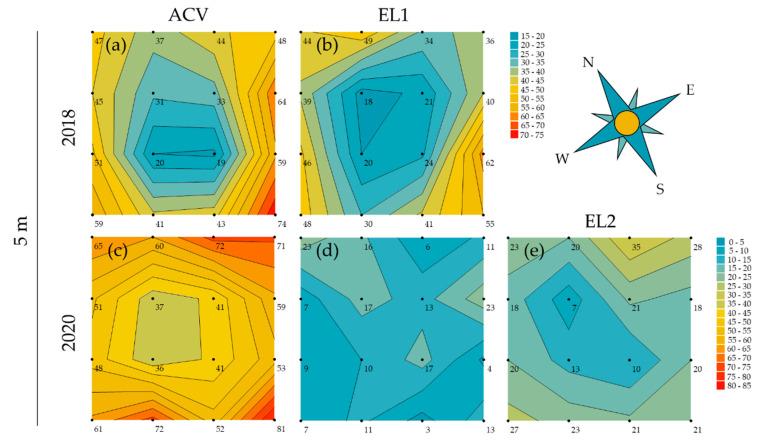
Contour plots for the trials in spring at 5 m inter-trap spacing. Each point of the 4 × 4 grids represents the pooled *D. suzukii* (male and female) trap catches for a trap position over the whole spring (May and June) trial period and the four replicate grids. The contour plots for (**a**) ACV and (**b**) EL1 in 2018 both show a clear depression in the centre of the grid. The plots are relatively symmetrical and there is no evident relation with the mean NNW winds. In 2020, the contour plots of (**c**) ACV and (**e**) EL2 again show a similar depression in the centre whereas this is not seen for (**d**) EL1. Additionally, here no evident relation with the mean wind direction (WSW) can be observed.

Contour plots were made for each trial per attractant to uncover or visualise spatial patterns in the trapping grids. This could be patterns related to trap interference, hence visualising what was analysed in Section 3.1. However, there could also be effects of wind direction or insect migration in the orchard. During spring, at 5 m inter-trap spacing, there was a trapping period of 9 and 10 weeks for 2018 and 2020, respectively. The contour plots show a clear depression in the centre of the grid and are relatively symmetrical for both ACV and EL1 in 2018 (Figure 3a,b). In 2020, the same is seen again for ACV, but not for EL1, while EL2 did show a clear depression in the centre (Figure 3c–e). Other than the expected circular pattern due to trap interference, no evident patterns were observed.

**Figure 4:**
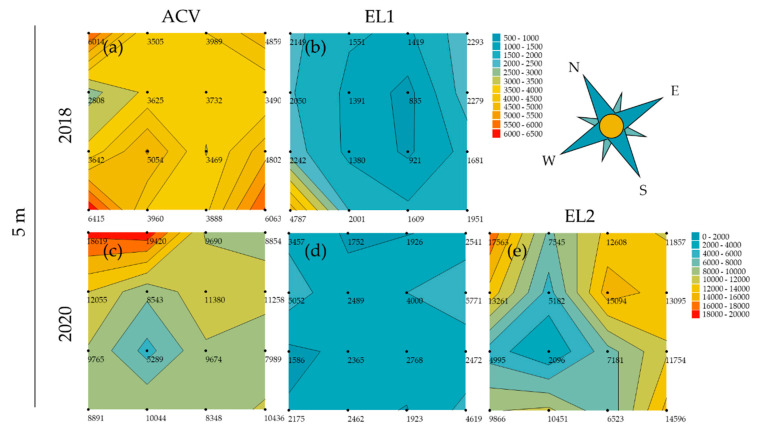
Contour plots for the trials in summer at 5 m inter-trap spacing. Each point of the 4 × 4 grids represents the pooled *D. suzukii* (male and female) trap catches for a trap position over the whole trial period (August) and the four replicate grids. The contour plots for (**a**) ACV and (**b**) EL1 in 2018 both show a clear depression in the centre of the grid and peaks on the corners. For both plots, the highest peak is on the most western (corner) trap position. The mean wind direction in this period was SW. In 2020, the contour plots of (**c**) ACV and (**e**) EL2 again show a depression in the centre, while this is not seen for (**d**) EL1. Here, for ACV, the plot is asymmetrical, with mainly one centre and one corner trap position causing the contrast. For both ACV and EL2 in 2020, the highest peak is seen on the most northern (corner) trap position and the least captures are observed on the same western centre trap position. Mean wind direction during this period was SSE.

The trials at 5 m inter-trap spacing in summer show, in 2018, clear depressions in the centre of the grids and peaks at the corners for both ACV and EL1 (Figure 4a,b), whereas in 2020, this depression is not clear for EL1, but only for ACV and EL2 (Figure 4c–e). For ACV, however, the plot is asymmetrical, with mainly one centre and one corner trap position causing the contrast. For both ACV and EL2 in 2020, the highest peak is seen on the most northern (corner) trap position and the least captures are observed on the same western centre trap position.

**Figure 5:**
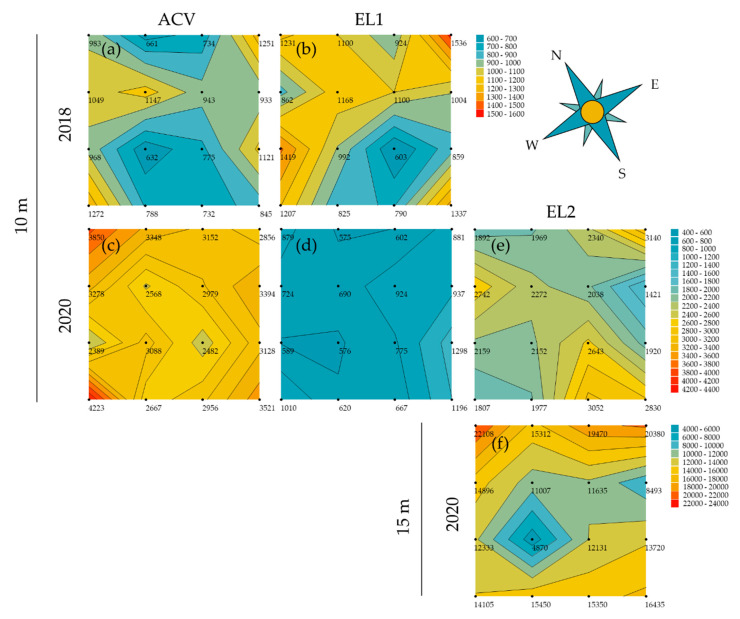
Contour plots for the trials in summer at 10 and 15 m inter-trap spacing. Each point of the 4 × 4 grids represents the pooled *D. suzukii* (male and female) trap catches for a trap position over the whole trial period (August/September) and the four replicate grids. The contour plots for (**a**) ACV and (**b**) EL1 in 2018 both show a similar depression in the southwestern centre trap positions. For EL1, there is a pattern of peaks on the corner trap positions; for ACV this is less evident. The mean wind direction in this period was SSW. In 2020, the contour plot of (**c**) ACV shows a depression in the centre and peaks on the corner trap positions. This pattern is not as clear for (**d**) EL1 and absent for (**e**) EL2. Mean wind direction during the corresponding period was SW. (**f**) The plot for EL2 at 15 m inter-trap spacing shows a clear depression in the centre with peaks on the corner trap positions. The mean wind direction was SSE during this trial.

The trials with 10 m inter-trap spacing (summer) in 2018 show lessevident trap interference patterns for both ACV and EL1 (Figure 5a,b). There is, for both attractants (for EL1 more so than for ACV), however, still a trend of lower trap catches in the centre traps and higher catches in the corner traps. The contour plots for the 10 m inter-trap spacing (summer) trial in 2020 show a depression in the centre and peaks on the corner trap positions for ACV; this trap interference pattern was less distinct for EL1 and absent for EL2 (Figure 5c–e). The plot for ACV has a low value (2389) for one of the west edge trap positions, which is due to a missing trap in one of the grids. It otherwise shows a rather circular pattern with the highest peaks on the north and west corner trap positions (Figure 5c). The mean wind direction during the corresponding period was SW.

The plot for EL2 at 15 m inter-trap spacing shows a clear trap interference pattern: depression in the centre with peaks on the corner trap positions (Figure 5f). The north and north-east trap positions hold the highest trap catches. The mean wind direction was E during this trial.

### 3.3. Attractant Performance

**Figure 6:**
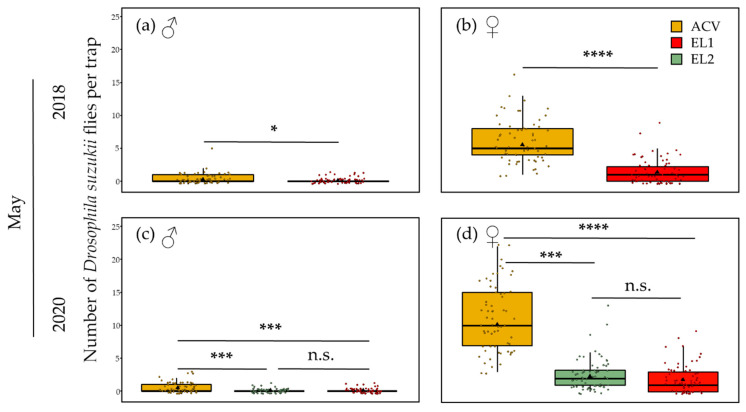
Number of *D. suzukii* flies per trap per sex and attractant in May. Jittered points and boxplots represent the 64 replicate traps and their distribution, respectively. Black triangles represent the estimated marginal mean number of *D. suzukii* flies per trap (GLMM). Lines connecting boxplots with asterisks above indicate the level of significance regarding the difference in the estimated marginal means of different attractants (GLMM, * *p* ≤ 0.05, ** *p* ≤ 0.01, *** *p* ≤ 0.001, **** *p* ≤ 0.0001, n.s.: not significant). (**a**) For the males in May 2018, regardless of the low proportion of overwintering males, significantly more flies were caught with ACV than with EL1. (**b**) For the females, the same is observed. (**c**,**d**) In 2020 for both males and females, ACV caught significantly more *D. suzukii* than both EL1 and EL2, with the latter two not significantly difference.

Early in spring, regardless of the attractant, *D. suzukii* trap catches were generally low and comprised mainly of females, with 23 and 22.1 times more females than males in ACV for May 2018 and 2020, respectively. For EL1 in May, there were 14.6 and 22.6 times more females than males in 2018 and 2020, respectively. EL2 in May 2020 had 35 times more females than males. In June, those ratios overall became smaller. In 2018 there were even more males caught than females: 1.15 times more for ACV and 1.4 times more for EL1. In June 2020, however, for ACV 7.3 times more females than males were caught, while for EL2 and EL1, this was 2.99 and 3.68 times, respectively. In May of both years, ACV caught significantly more *D. suzukii* flies than both EL1 and EL2 did. In 2018, the estimated marginal mean number of *D. suzukii* males for ACV was 0.24, which was significantly higher (GLMM: z-ratio = 2.14, *p* = 0.03, *n* = 64) than the 0.09 value of EL1 (Figure 6a). The estimated marginal mean number of females for ACV was 5.56, which was significantly higher (GLMM: z-ratio = 8.26, *p* < 0.0001, *n* = 64) than the 1.38 value for EL1 (Figure 6b). In 2020, the estimated marginal mean number of males for ACV was 0.46, which was significantly higher than the 0.07 and 0.08 for EL2 (GLMM: z-ratio = 3.78, *p* = 0.0005, *n* = 64) and EL1 (GLMM: z-ratio = 3.67, *p* = 0.0007, *n* = 64), respectively (Figure 6c). For the females, this mean value for ACV was 10.18, which is significantly higher than the 2.33 value for EL2 (GLMM: z-ratio = 7.46, *p* < 0.0001, *n* = 64) and 1.80 for EL1 (GLMM: z-ratio = 8.57, *p* < 0.0001, *n* = 64) (Figure 6d).

**Figure 7:**
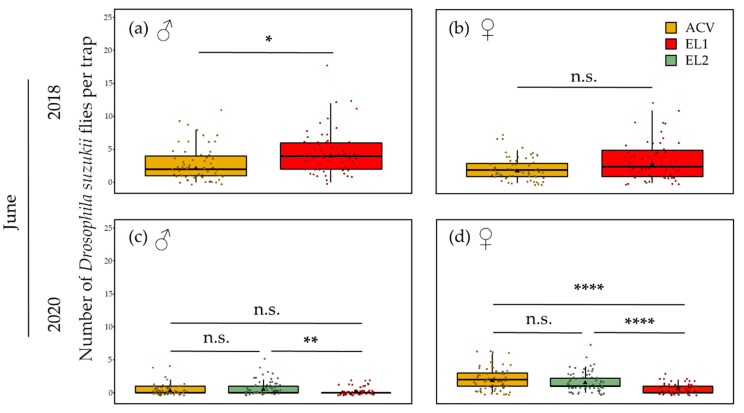
Number of *D. suzukii* flies per trap per sex and attractant in June. Jittered points and boxplots represent the 64 replicate traps and their distribution, respectively. Black triangles represent the estimated marginal mean number of *D. suzukii* flies per trap (GLMM). Lines connecting boxplots with asterisks above indicate the level of significance regarding the difference in the estimated marginal means of different attractants (GLMM, * *p* ≤ 0.05, ** *p* ≤ 0.01, *** *p* ≤ 0.001, **** *p* ≤ 0.0001, n.s.: not significant). (**a**) In June 2018, significantly more males were caught with EL1 than with ACV. (**b**) For the females, the difference was not significant. (**c**) In 2020, EL2 caught significantly more male *D. suzukii* flies than EL1. The number of males caught by ACV did not significantly differ from that of both EL1 and EL2. (**d**) For the females in June 2020, ACV and EL2 did not significantly differ in fly catches, but both caught significantly more flies than EL1.

In June, the synthetic kairomones EL1 and EL2 became competitive with ACV. In 2018, EL1, with an estimated mean number of male *D. suzukii* flies of 4.00, had a significantly higher (GLMM: z-ratio = −2.06, *p* = 0.04, *n* = 64) catch than ACV with a mean value of 2.10 (Figure 7a). For the females, there was no significant difference (Figure 7b). In 2020, for both males and females, ACV (estimated marginal mean **♂**: 0.25 and ♀: 1.84) and EL2 (**♂**: 0.49 and ♀: 1.48) were not significantly different (Figure 7c,d), whereas for the males the estimated marginal mean was significantly higher (GLMM: z-ratio = 3.33, *p* = 0.0025, *n* = 64) for EL2 than for EL1 (0.15). For the females, the means were significantly higher for both ACV (GLMM: z-ratio = 5.83, *p* < 0.0001, *n* = 64) and EL2 (GLMM: z-ratio = 4.64, *p* < 0.0001, *n* = 64) than that for EL1 (0.56).

**Figure 8:**
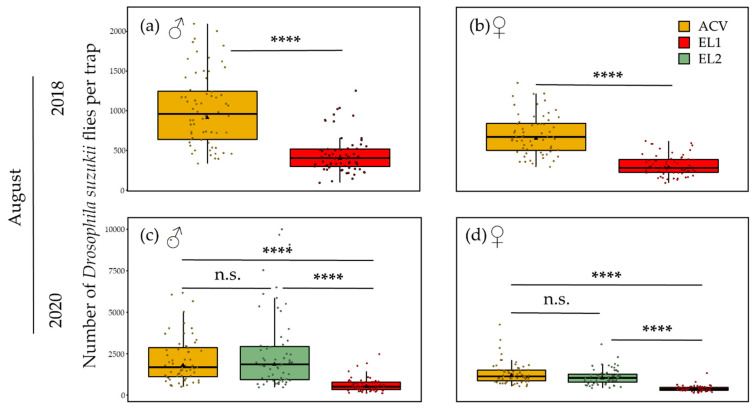
Number of *D. suzukii* flies per trap per sex and attractant in August. Jittered points and boxplots represent the 64 replicate traps and their distribution, respectively. Black triangles represent the estimated marginal mean number of *D. suzukii* flies per trap (GLMM). Lines connecting boxplots with asterisks above indicate the level of significance regarding the difference in the estimated marginal means of different attractants (GLMM, * *p* ≤ 0.05, ** *p* ≤ 0.01, *** *p* ≤ 0.001, **** *p* ≤ 0.0001, n.s.: not significant). (**a**) In August 2018, significantly more males were caught with ACV than with EL1. (**b**) For the females, the same was observed. (**c**,**d**) In 2020, for both males and females, ACV and EL2 did not significantly differ in fly catches, but both caught significantly more flies than EL1.

In August, the population size of *D. suzukii* was large, which translated in high trap catches. In this sour cherry orchard after harvest, there were more males caught than females in both 2018 and 2020. In 2018, there were 1.4 times more males than females for ACV and 1.5 times more for EL1. In 2020, there were 1.6 times more males than females for ACV and 2 and 1.7 times more for EL2 and EL1, respectively. ACV and EL2 were, in summer, not significantly different in terms of catches and were both better performing than EL1. For the males in 2018, ACV had an estimated marginal mean of 913.87, which was significantly higher (GLMM: z-ratio = 4.88, *p* < 0.0001, *n* = 64) than the mean value of EL1 (398.44) (Figure 8a). Similarly, for the females, the mean of 635.62 for ACV was significantly higher (GLMM: z-ratio = 6.53, *p* < 0.0001, *n* = 64) than that of EL1 (263.80) (Figure 8b). For both the males and females in 2020, ACV (estimated marginal mean **♂**: 1782.18 and ♀: 1114.00) and EL2 (**♂**: 1858.34 and ♀: 948.96) were not statistically different (Figure 8c,d). For the males, the means were significantly higher for both ACV (GLMM: z-ratio 4.76=, *p* < 0.0001, *n* = 64) and EL2 (GLMM: z-ratio = 4.92, *p* < 0.0001, *n* = 64) than for EL1 (503.96). For the females, similarly, the means were significantly higher for both ACV (GLMM: z-ratio = 11.67, *p* < 0.0001, *n* = 64) and EL2 (GLMM: z-ratio = 10.26, *p* < 0.0001, *n* = 64) than for EL1 (294.43).

## 4. Discussion

This two-year study investigated the potential of a dry (i.e., synthetic lures in a controlled release dispenser), non-sticky trap for mass trapping *D. suzukii,* with pure ACV being selected as a reference attractant. In 2018, only EL1 was compared to ACV and in 2020, the generally better performing EL2 was added. Trap interference in 4 × 4 minigrids was used to estimate the attraction radius and thus mass trapping potential [94]. In both 2018 and 2020, first an inter-trap spacing of 5 m was tested in spring (cherry cropping) as well as in summer (after cherry harvest yet relevant for later susceptible crops). In both years, in summer also, an inter-trap spacing of 10 m was tested and, in 2020, even 15 m spaced traps were tested for EL2.

In May of both years, regardless of the attractant, traps captured about 20 times more females than males. In June 2020, this female bias started to decrease, between 3 and 7 times more in females than males. In June 2018, there was already a slight male bias (between 1.2 and 1.4 times more males). This might be explained by the earlier phenology of *D. suzukii* in 2018 than in 2020 (based on temperature/degree day-dependent phenology simulations and monitoring observations, data not shown). In August, the trap catches regardless of attractant, were always male biased (between 1.4 and 2 times more males). Female-biased trap counts in winter and early spring, a shift to male biased counts in late spring and a clear male bias in summer and autumn have been commonly observed [6,8,100,101,102,103,104], with the strongest reported female spring bias being in cherry orchards [105], as in the present study. Although trap catches can yield a distorted representation of *D. suzukii* population dynamics [70], the lower numbers of males caught in spring are most likely linked to their poorer cold tolerance and thus lower winter survival [8,106,107,108]. The higher proportion of trapped males in August could be the result of migrated reproductive females due to the absence of fresh fruit oviposition substrates in cherry orchards at that time and/or the effects of physiology or sex on the olfactory preferences of *D. suzukii* [70,109,110]. In general, ACV was observed to be far less selective than EL1 and EL2, regardless of seasonal timing, with the synthetic lures even highly selective in terms of Drosophilidae. The observations of numerous probable *Drosophila* parasitoids in ACV in the spring were remarkable, which is, however, in line with earlier observations for ACV in California [111].

During spring, when spacing the traps at 5 m, there were clear differences between the results of beginning of the spring (~May) and a month later (~June). In May over both years tested, ACV consistently showed trap interference: significantly more catches in corner than centre traps, with a mean corner: centre ratio of 2.99 (2018) or 1.94 (2020). Despite trap catches still being very low this early in spring, the corner traps attract double or triple the amount of *D. suzukii* flies. The synthetic dry lures captured about five times less than ACV did in this period. EL1 did not show interference and had an accordant mean corner: centre ratio of about 1 for both years in May. Conversely there were significantly less flies captured in corner traps than in centre traps loaded with EL1 in May 2020. This may be the result of non-overlapping attraction radii, but a general attraction towards the trapping grids, concentrating the flies more in the centre, with higher chances of captures there (spill-over effect [112]). EL2 showed a mean corner: centre ratio of 2.44, but the difference in counts between corner and centre traps was not significant. In June, this difference between ACV and the experimental lures diminished, with EL1 in 2018 catching more *D. suzukii* males than ACV. In June 2020, EL2 and ACV performed similarly and significantly outperformed EL1. The differences in catching performances of EL1 between June 2018 and 2020 might be due to the fact that this lure seems more attractive for *D. suzukii* summer generations, which appeared earlier in 2018 compared to 2020. In June 2018, trap interference was significant for both EL1 as ACV with high corner: centre ratios of, respectively, 3.60 and 2.96. This interference pattern was also apparent on the contour plots for spring, with EL1 having compensated its low captures from May. Similar numbers of *D. suzukii* were obtained per attractant in May 2018 and May 2020. In June 2020, however, lower numbers were captured than in June 2018.

In June 2020, only for EL2, significant trap interference was observed with a mean corner: centre ratio of 2. While ACV showed a similar mean corner: centre ratio, trap interference was not found significant. In line with the low trap captures for EL1 in June 2020, trap interference for this lure was not seen in that period, the latter possibly being the result of the former (i.e., small sample sizes). On the contour plots of spring 2020, the above is confirmed: no pattern for EL1, but clear patterns of interference for ACV and, despite its limited captures in May, also for EL2.

Our data thus indicate that the synthetic lures are competitive with ACV from June onwards, with EL2 performing better than EL1. In the spring, both ACV and the synthetic lures show trap interference at 5 m inter-trap spacing. Suckling et al. (2015) stated that in 4 × 4 minigrids the corner: centre trap ratio is predicted to peak at about 2.62 (when the inter-trap spacing relative to the attraction radius (d/r) ≈ 0.6) (Figure S1). The present study shows that, for ACV in 2018, this maximum was reached (with even higher values) and that the attraction radius for ACV in spring was about 8.3 m (5 m/0.6) [94]. Considering the theory that for mass trapping d/r should be smaller than $\sqrt{2}$ (overlap of diagonally neighbouring traps), this may indicate that ACV (in the present trap design and situation) should not be spaced more than 11.8 m. In spring 2020, for ACV corner: centre ratios of about 2 were obtained and, for May, this interference was significant. According to the aforementioned model, a ratio of 2 corresponds to either an estimated d/r ≈ 1 or an estimated d/r ≈ 0.25 (Figure S1) [94]. As no other inter-trap spacings but 5 m were tested in spring, it cannot be concluded which trap spacing corresponds to a maximal corner: centre trap ratio at this seasonal timing. Nevertheless, based on the totality of other data obtained for ACV in this study, it can reasonably be assumed that the inter-trap spacing (d) of 5 m was not smaller than the attraction radius (r), excluding the possibility of d/r ≈ 0.25. Consequently, with an estimated d/r ≈ 1, in May 2020, ACV showed an attraction radius of about 5 m and the maximal trap distance for mass trapping in this situation would be 7.1 m. In spring, the experimental dry lures, tested at 5 m, only showed significant trap interference in June, with EL1 in 2018 reaching a mean corner: centre ratio of 3.60. This value is higher than the predicted maximum of 2.62 (Figure S1), which corresponds with an attraction radius of 8.3 m and a maximal trap distance for mass trapping of 11.7 m. In June 2020, EL2 showed significant trap interference with a mean corner: centre ratio of 2, which again corresponds with an attraction radius of 5 m and maximal trap distances of 7.1 m for mass trapping.

In the summer of both years, ACV had significantly higher trap catches than EL1. On the other hand, EL2 performed very similarly to ACV in this period. The trap counts in the summer of 2020 were generally higher than in 2018. During summer (August), experiments were conducted at both 5 m and 10 m inter-trap distance. At 5 m in August 2018, EL1 showed significant trap interference with a mean corner: centre ratio of 2.52. This was clearly higher than when this lure was tested at 10 m inter-trap spacing that same month, for which, despite significant trap interference, a rather low mean corner: centre ratio of 1.34 and a less obvious pattern on the contour plot were observed. According to the model [94], a ratio of 2.52 corresponds to either an estimated d/r ≈ 0.72 or d/r ≈ 0.48 (Figure S1), corresponding to an estimated attraction radius of either 6.9 or 10.4 m, respectively, and required trap distances for mass trapping between 9.8 and 14.7 m. At 5 m a trap competition pattern is also seen for both EL1 and ACV on the contour plots (Figure 4a,b), with EL1 showing a greater relative depression in the centre. Thus, despite lower trap catches, EL1 showed a larger mean corner: centre ratio than ACV (1.46). This may seem contradictory, but it is, however, known that *D. suzukii*’s physiological status can influence its olfactory preferences [70,110]. Therefore, it is possible that the attraction of EL1 for a specific fraction of the population (e.g., flies of a specific age group or feeding status) is larger than that of ACV for another (yet larger) fraction of the population. As in June of that year, only EL2 showed significant trap interference in August 2020 at 5 m with a mean corner: centre ratio of 1.69. In the trial with 10 m inter-trap spacing in that month, the trap interference was no longer significant, with a mean corner: centre ratio of only 1.17. Again, using the aforementioned model, a ratio of 1.69 corresponds to either an estimated d/r ≈ 1.15 or an estimated d/r ≈ 0.15 (Figure S1). Given the fact that, for the 10 m inter-trap spacing, no trap interference was observed, the former is clearly more evident. With d/r ≈ 1.15 for 5 m inter-trap spacing for EL2 in the summer of 2020, the attraction radius can be estimated to be about 4.3 m and maximal trap distance for mass trapping to be about 6.2 m. Thus, in summer the experimental lures seem to have a larger attraction radius than ACV. The latter showed, in the summer of 2020, no significant trap interference at 5 m inter-trap spacing, but it did in the trial at 10 m. In that trial, ACV had a mean corner: centre ratio of 1.40 and a and a clear trap interference pattern on the contour plot (Figure 5c). According to the model [94], a ratio of 1.40 corresponds to either an estimated d/r < 0.1 (which is extremely unlikely in this case) or an estimated d/r ≈ 1.35 (Figure S1). Hence, an attraction radius of 7.4 m and a maximal distance for mass trapping of 10.5 m could be estimated for ACV based on this trial.

When testing at an even larger inter-trap spacing of 15 m for EL2 in September 2020, this dry lure remarkably showed significant trap interference, with a mean corner: centre ratio of 2.06. It also showed a clear interference pattern on the contour plot, with a depression in the centre and peaks on the corner trap positions. Although the tests at 5 m, 10 m and 15 m were conducted at the same location and within two months, they were performed in sequence and thus subjected to varying environmental conditions and *D. suzukii* population characteristics. As seen on the contour plots (Figure 5e,f), the cumulative trap catches were about 7 times higher in the trial at 15 m than the trial at 10 m, although the latter lasted three weeks, whilst the former lasted only two. The higher trap interferences obtained in this period might be the result of the decreasing availability of suitable food/oviposition sources, making EL2 more attractive, and this for a considerably larger *D. suzukii* population (and hence larger sample sizes). The corner: centre ratio of 2.06 for EL2 at 15 m corresponds with an estimated attraction radius of 15 m and hence a minimal trap distance for mass trapping of 21.2 m in these conditions.

*Drosophila melanogaster* displays upwind anemotaxis during flight and uses similar odour plume-tracking mechanisms as moths [113,114]. However, this fruit fly also uses (down)wind assisted dispersal, for long-distance flights, at higher windspeeds [115]. Considering the contour plots of the present study and the mean wind directions during the trials, peak trap catches appear to effectively occur on the line of the mean wind direction (except for Figure 5f). When mean windspeeds are highest, peaks appear to be related to downwind migration (Figure 4a,b and Figure 5c), whereas at lower wind speeds, peaks may be due to upwind plume-tracking (Figure 4c,e).

There are few studies on the mass trapping of *D. suzukii*. Kanzawa (1939) reported mass trapping using bottles with fermentation products as an effective control measure in Japanese cherry orchards using 30 to 40 traps per ha [25]. As the latter study was also conducted during the cherry season, it can be compared with the spring situation of the present study, where a higher density (75 to 200 traps/ha) was estimated to be required. Hampton et al. (2014) performed a field trial on perimeter trapping (1.8 m between traps) using cups with a fermenting bait (water, ACV, baker’s yeast and flour) in small replicate blueberry plots. The traps did not effectively control *D. suzukii* in this trial and the authors pointed out a spill-over effect, with traps attracting flies from nearby plots, while not effectively capturing or retaining them. This phenomenon was probably strong because of the small plots and perimeter trapping approach. It was observed that the percentage of infested fruits declined at a distance of 5.5 m from the trap, suggesting that the trap attracted *D. suzukii* up to about 5.5 m [21]. This corroborates the estimations of the attraction radius being between 5 and 10.5 m for ACV in the present study. Spies and Liburd (2019) conducted a similar field trial on perimeter trapping (5 m between traps) using cups with a fermentation bait (RIGA^®^ AG: cider vinegar, red wine, sugar, and berry juice) in small replicate plots in blueberry. Significantly less *D. suzukii* flies were caught in the central monitoring traps in the small plots with perimeter trapping than in control plots, suggesting that these traps at 5 m distance provided a working perimeter and/or that the perimeter traps competed with the inner trap. This is also in line with the findings for the ACV bait in the summer trials of the present study. Wallingford et al. (2018) used trapping (pull) with red sticky sphere traps baited with Scentry Spotted Wing Drosophila Lure (Scentry Biologicals, Billings, MT, USA) in a field trial on a push-pull strategy in small (4 × 4 m) potted blueberry plots. The “pull only” plots, having a trap on each corner of the 4 by 4 m plot, showed low trapping efficiency and higher fruit infestation than the control. Here, again the importance of a large covering trapping grid with an adequate trap spacing (and optimal trapping mechanism) is emphasised to combat this spill-over effect. For cropping systems where *D. suzukii* is not found overwintering inside the plot, but is known to enter from neighbouring habitats, the estimated attraction radii from the present study can be a base in the development of effective perimeter trapping strategies. Perimeter trapping, however, may hold the risk of *D. suzukii* passing the barrier of traps by passive (wind) dispersal.

## 5. Conclusions

The quantification of trap interference in 4 × 4 minigrids can be considered as a suitable method for investigating the feasibility of mass trapping *D. suzukii* for a combination of trap design, attractant and environmental conditions (crop, seasonal timing, climate, etc.). The method enables to estimate a range of trap densities for further studies as well as to compare the mass trapping potential of attractants.

In this study, traps designed for dry trapping (i.e., traps without drowning solution, but with synthetic lure dispensers and a killing agent inside) were evaluated while baited with two experimental dry lures and pure ACV as a reference attractant. In May in sour cherry, when *D. suzukii* populations are still small and comprise mostly of overwintering females, ACV shows the most potential for mass trapping with both consistently the highest trap counts and significant trap interference. From June onwards, the experimental dry lures show equal or better results than ACV. The estimated trapping parameters from the present study suggest that, during spring, depending on the attractant and the environment (e.g., climate affecting *D. suzukii* phenology) a density of about 75 up to 200 traps/ha would result in grids of traps with contiguous attraction radii. During summer, tested in this paper in a cherry orchard after harvest, the estimated required minimal trap densities were found to be between about 90 and 300 traps/ha. In September, the synthetic dry lure EL2, in theory, would only require about 25 traps/ha under the conditions of that trial. These trap density ranges are rather wide, but they clearly indicate the potential of mass trapping *D*. *suzukii,* also with workable, long-lasting lures. Moreover, the results of this study are a starting point for further field research on “dose-finding” and control efficacy. Eventually, mass trapping could become part of the integrated *D. suzukii* management, contributing to the reduction in broad-spectrum insecticide applications.

## Data Availability

The data presented in this study are available on request from the corresponding author.

## Supplementary Materials

The following supporting information can be downloaded at: https://www.mdpi.com/article/10.3390/insects13030240/s1, Figure S1: Simulated data points and LOESS regression based on the model of Suckling et al. (2015) for the relation between d/r (trap spacing relative to attraction radius) and the ratio of corner over centre trap catches in a 4 × 4 minigrid of traps.
