# Supplementary material for: Mass Trapping Drosophila suzukii, What Would It Take? A Two-Year Field Study on Trap Interference
Source: Insects. 2022 Feb 28;13(3):240. doi: 10.3390/insects13030240 (PMC8953694; doi:10.3390/insects13030240)
Supplement: Supplementary file 1 [file insects-13-00240-s001.zip › insects-1601101-supplementary.pdf]

insects

MDPI

Supplementary Materials

Mass Trapping *Drosophila suzukii*, What Would It Take? A Two-Year Field Study on Trap Interference

Rik Clymans, Vincent Van Kerckvoorde, Tom Thys, Patrick De Clercq, Dany Bylemans and Tim Beliën

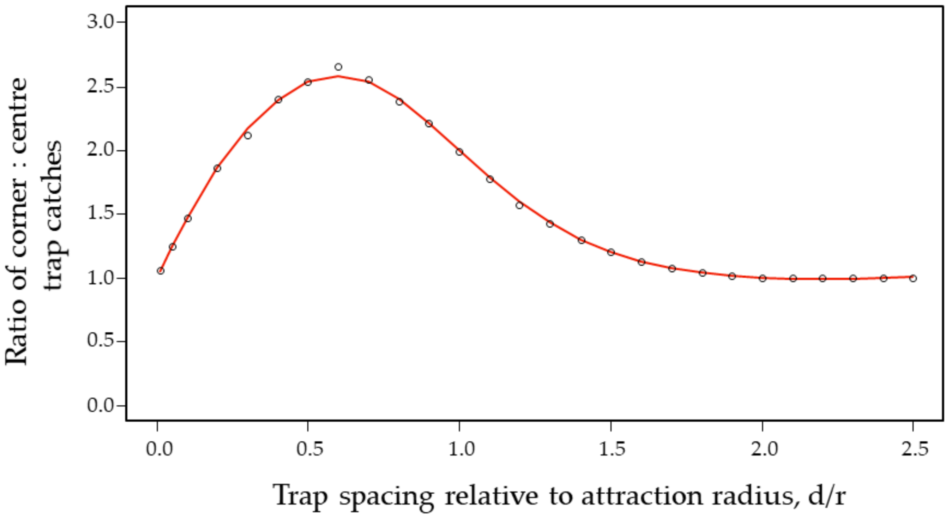

Y	X
1,05	0,01
1,26	0,05
1,47	0,1
1,83	0,2
2,18	0,3
2,41	0,4
2,56	0,5
2,60	0,6
2,55	0,7
2,33	0,8
2,22	0,9
1,97	1
1,76	1,1
1,58	1,2
1,43	1,3
1,31	1,4
1,20	1,5
1,13	1,6
1,08	1,7
1,04	1,8
1,02	1,9
1,00	2
1,00	2,1
1,00	2,2
1,00	2,3
1,00	2,4
1,00	2,5

Figure S1. Simulated data points and LOESS regression based on the model of Suckling et al. (2015) for the relation between d/r (trap spacing relative to attraction radius) and the ratio of corner over centre trap catches in a 4 × 4 minigrid of traps.
